# Mate choice in sticklebacks reveals that immunogenes can drive ecological speciation

**DOI:** 10.1093/beheco/arx074

**Published:** 2017-06-01

**Authors:** Demetra Andreou, Christophe Eizaguirre, Thomas Boehm, Manfred Milinski

**Affiliations:** a Max Planck Institute for Evolutionary Biology, Department of Evolutionary Ecology, August- Thienemann- Str. 2, D-24306, Ploen, Germany,; b Department of Life and Environmental Science, Faculty of Science and Technology, Talbot Campus, Poole, BH12 5BB, UK,; c GEOMAR| Helmholtz Centre for Ocean Research, Department of Evolutionary Ecology of Marine Fishes, D-24105, Kiel, Germany,; d Present address: School of Biological and Chemical Sciences, Queen Mary University of London, Mile End Road, London E1 4NS, UK, and; e Max Planck Institute of Immunobiology and Epigenetics, Department of Developmental Immunology, Stuebeweg 51, D-79108 Freiburg, Germany

**Keywords:** ecological speciation, magic trait, major histocompatibility complex, mate choice, parasite resistance

## Abstract

Adaptation to ecologically contrasting niches can lead to the formation of new species. Theoretically, this process of ecological speciation can be driven by pleiotropic “magic traits” that genetically link natural and sexual selection. To qualify as a true magic trait, the pleiotropic function of a gene must be reflected in biologically relevant mechanisms underlying both local adaptation and mate choice. The immune genes of the major histocompatibility complex (MHC) contribute to parasite resistance and also play a major role in sexual selection. Hence, the MHC may encode a candidate magic trait. Using diverging 3-spined stickleback populations from a connected lake–river habitat, we show with mate choice experiments in a flow channel that polymorphic MHC genes probably underlie assortative mating with respect to particular habitat-adapted ecotypes, potentially resulting in reproductive isolation. By manipulating olfactory cues in controlled experiments, we show that female sticklebacks employ MHC-dependent male olfactory signals to select mates with which they can achieve a habitat-specific MHC gene structure that optimally protects their offspring against local parasites. By using MHC-based olfactory signals, females thus select individuals of their own population as mates. Our results demonstrate how mate choice and parasite resistance may be functionally linked. These findings suggest that MHC genes are pleiotropic and encode a true magic trait of biologically significant effect.

## Lay Summary

The role of sexual selection in driving speciation is supported by correlative links between various traits under sexual selection and speciation. However, there is little evidence on the candidate genes involved in mate choice enabling speciation. Experiments on stickleback mate choice highlight the polymorphic immunogenes of the Major Histocompatibility Complex (MHC) as a true “magic trait”: the MHC drives both habitat-specific assortative mate choice and local adaptation—ultimately speciation.

## INTRODUCTION

Ecological speciation results from the evolution of reproductive isolation as a result of adaptation to divergent ecological niches (Schluter and Conte 2009). Natural selection, however, is not the only fuel for speciation as divergent sexual selection can also mediate and/or reinforce population differentiation (Coyne and Orr 2004; Lande 1981, reviewed by Ritchie 2007). The evolution of divergent sexual selection may stem from adaptation to diverging ecological conditions, which can then lead to divergence in sexual traits, sensory systems and mating preferences (Maan and Seehausen 2011). This pattern may result in locally adaptive phenotypes and genotypes (Eizaguirre et al. 2012a). In order for local adaptation to be maintained across sexual reproduction events (i.e. recombination), the mate choice mechanism must preserve the linkage disequilibrium between locally co-adapted genes or gene complexes. In doing so, the choosing sex might consider a number of cues, such as visual, acoustic, and olfactory cues, in selecting their mate (Boughman 2001; Candolin 2003).

Adaptive divergence is one of the main pillars of ecological speciation (Coyne and Orr 2004). This process is facilitated if selection acts on so-called “magic traits” which ensure that an association between divergent selection and non-random mating cannot be broken by genetic recombination (Gavrilets 2004; Thibert-Plante and Gavrilets 2013). A true magic trait must fulfill 3 criteria. First, the magic trait, and not a correlated trait (that is, a trait controlled by different genes), must be subject to divergent selection. Second, the magic trait, and not a correlated trait, must support non-random mating. Third, the effect of a magic trait should be of ecologically relevant magnitude. Hence, to qualify as a true magic trait, the pleiotropic function of a gene must be reflected in biologically relevant mechanisms underlying both local adaptation and mate choice (Haller et al. 2012; Servedio et al. 2011).

While relative contributions of reproductive barriers have been estimated (e.g. Nosil et al. 2005, Kay 2006) and even though several studies have linked a given selective pressure to reproductive isolation (e.g. diet [Snowberg and Bolnick 2008]; predation [Reynolds and Fitzpatrick 2007]; parasites [Eizaguirre et al. 2009a]; sensory environment [Seehausen et al. 2008]), the genes underlying adaptive divergent phenotypes remain to be identified (but see Seehausen et al. 2008). Parasites represent a dynamic biotic selective pressure for their hosts (Hamilton et al. 1990; Poulin 2007) and can mediate both natural and sexual divergent selection (Buckling and Rainey 2002; Eizaguirre and Lenz 2010; Eizaguirre et al. 2009a; Thompson and Cunningham 2002). The identification of the major histocompatibility complex (MHC) as a dominant factor determining the response to pathogens and parasites provides a potential functional genetic link for ecological speciation (Eizaguirre et al. 2009b). The MHC genes encode a suite of structurally related yet distinct molecules, which are present in all jawed vertebrates and function as an important component of the adaptive immune system regulating immune homeostasis and resistance against parasites and diseases (Janeway et al. 2005). In particular, parasite resistance has been directly linked to the evolution of MHC allele frequencies (Eizaguirre et al. 2012b) and their local adaptation (e.g. Babik et al. 2008; Eizaguirre et al. 2012a; Loiseau et al. 2009; Wegner et al. 2003), illustrating how parasites mediate divergent selection and impact the evolutionary trajectories of their hosts. Furthermore, it has been demonstrated that evolved optimal individual MHC diversity of the host can maximize parasite resistance (Milinski 2006; Wegner et al. 2003), fitness relevant trait (Buchholz 2004; Stiebens et al. 2013), survival (Kalbe et al. 2009), and thus lifetime reproductive success (Eizaguirre et al. 2009b; Wegner et al. 2008).

In addition to their immune functions, MHC genes also play a major role in sexual selection (Milinski et al. 2005; Reusch et al. 2001a). It is now clear that MHC-based mate choice is common (but not universal, Richardson et al. 2005) in the vertebrate kingdom as evidence exists for mammals (e.g. mice [Yamazaki et al. 1976]), fish (e.g. Forsberg et al. 2007), birds (e.g. Bonneaud et al. 2006; Griggio et al. 2011), primates (e.g. Schwensow et al. 2008), and human (e.g. Chaix et al. 2008; Wedekind et al. 1995).

For our study, we used the 3-spined stickleback (*Gasterosteus aculeatus* L.) as the model organism. This fish has become a prime example for adaptive radiation (Bell 1994), which resulted in parallel lake–river ecotypes all over the Northern Hemisphere (Berner et al. 2009; Bolnick et al. 2009; Hendry 2009; Hendry et al. 2002; Ravinet et al. 2013; Reusch et al. 2001b), for review see (Hendry 2009).

In addition to color (e.g. Milinski and Bakker 1990), sticklebacks choose mates using their sense of smell (Eizaguirre et al. 2011; Haberli and Aeschlimann 2004; Heuschele et al. 2009; McLennan 2004; Milinski et al. 2005; Milinski et al. 2010; Rafferty and Boughman 2006; Reusch et al. 2001a; Sommerfeld et al. 2008). One function of olfactory mate choice is improving resistance of the offspring against infectious diseases (see review by (Milinski 2006). Female sticklebacks use reference to self-MHC to be able to choose males that contribute optimally complementary types of MHC alleles to their progeny to maximize parasite resistance (Milinski et al. 2005; Reusch et al. 2001a). Such olfactory-based mate choice can be predictably modified by the addition of synthetic MHC-ligand peptides to a male’s natural MHC odor (Milinski et al. 2005). This finding suggests that the sequence composition of peptide ligands, once released from their specific peptide-MHC complexes, can be decoded to reveal the MHC molecule’s structure. Because peptides and MHC molecules fit together in a lock and key mode, the peptide sequence can be read as a proxy of MHC allele identity. In this way, peptides serve as the natural MHC odor signal.

The MHC signal is released by male sticklebacks; however, the shedding of peptide–MHC complexes potentially compromises immune function, selecting against unconditional use of these costly signals (Milinski et al. 2010). Importantly, the MHC signal is not released in isolation. It needs to be validated by a “validation factor” (Milinski et al. 2010; Sommerfeld et al. 2008). The significance of a validation factor becomes obvious when considering the generality of the mate choice mechanism. If all vertebrates indeed release peptides to attract mates, then a gravid female stickleback would risk, without a concomitant validation factor, to be attracted by a displaying male predatory fish. The peptide signal, which conveys information about the MHC structure of the individual, thus needs to be accompanied by an additional odor cue providing information beyond individuality, i.e. relating to species of the sender. Stickleback males begin producing this validation factor under spring conditions when they do not yet maintain a nest (Milinski et al. 2010) whereas the MHC signal is sent only when the males maintain a nest and court females (Sommerfeld et al. 2008).

Three-spined sticklebacks have more parasite species in lakes than in rivers (Eizaguirre et al. 2011; Feulner et al. 2015; Kalbe et al. 2002; Wegner et al. 2003) explaining the higher number of MHC alleles per fish in lakes. Some parasites are found in both habitats but there are many that are exclusive either to lake or to river (Eizaguirre et al. 2011). Also, MHC alleles largely differ between lake and river sticklebacks. A field experiment with laboratory-bred F2 sticklebacks that had either the lake or the river MHC were exposed in cages to the natural local parasites in lakes and rivers, respectively, for 9 months, proved that the MHC-genotypes are locally adapted to best resist local lake and river parasites, respectively (Eizaguirre et al. 2012b). Because females prefer males from their own habitat when given the choice between the odor of a river and a lake male (Eizaguirre et al. 2011), there must be a habitat-specific part of the odor signal. This can be part of the MHC signal or the male signal validating the MHC signal (Milinski et al. 2010) might be habitat-specific. The present study provides a decisive test the logic of which is presented below.

Each MHC peptide ligand, i.e. the MHC odor cue (Leinders-Zufall et al. 2004; Milinski et al. 2005), signals the possession of one specific MHC allele of the sender. However, MHC peptides in solvent compared with only solvent are not attractive for a female stickleback. Only when combined with the natural signal of a displaying male, peptides are counted as additional MHC alleles (Milinski et al. 2005). Thus, the natural peptide signal must be accompanied by an additional odor cue, i.e. the validation factor. It might be species-specific or even habitat-specific. We developed a technique to test whether a male sends only the validation factor or the full signal including the MHC component. Sending the validation signal precedes sending the MHC signal by a few weeks (Sommerfeld et al. 2008). By using a male that is already optimally MHC-fitting for a specific female, we add a mix of 4 synthesized MHC peptide ligands to its natural signal. If he already sends the complete optimal signal, the added 4 peptides, counted as additional alleles, turn his signal into a super-optimal one to be avoided (Milinski et al. 2005). If he does not yet send the MHC signal but only the validation factor, the spiked side would signal a male with 4 MHC alleles to be preferred to the un-spiked side where no MHC allele is signaled (Milinski et al. 2010). If the male does not even send the validation signal, both options would be equally unattractive. We used this method in the present study to verify that an experimental male does not send the MHC signal. If there would be a preference for the “same habitat” male, the habitat cannot have been detected from the MHC signal but from something else, e.g. the validation factor.

To examine whether a magic trait(s) underlies this adaptive radiation, we took advantage of the post-glacial divergence of lake–river ecotypes found in Northern Germany (Eizaguirre et al. 2011; Reusch et al. 2001b). Earlier studies had established that lake and river fish harbor different parasite communities (Kalbe et al. 2002; Wegner et al. 2003) and exhibit different, locally adapted MHC allele pools (Eizaguirre et al. 2012a; Eizaguirre et al. 2011). Here, we focus on the hypothesis that MHC genes encode a magic trait with pleiotropic roles in habitat-specific parasite resistance and choice for sympatric males. Our hypothesis makes 2 predictions. First, the diversity of MHC alleles and, consequently, the MHC signal is specific for and distinguishable between populations. Second, the male validation factor is invariant between populations; indeed, if the validation factor would be population specific, the validation factor would carry the required information for choosing a sympatric mate and the MHC genes would not have to be invoked as a magic trait. In all experiments we allow gravid females to choose between 2 odors of potential mates using an established 2-armed flow channel design (Milinski et al. 2005; Milinski et al. 2010; Reusch et al. 2001a; Sommerfeld et al. 2008).

## METHODS

### Animals

During winter, 3-spined sticklebacks were caught from 2 populations, the lake Grosser Plöner See (54°9′ 21.16° N, 10°25′50.14° E) and the river Malenter Au (54°12′ 16.19° N, 10°33′ 32.93° E) in Northern Germany. Despite being geographically connected, these 2 populations exhibit reduced gene flow (Eizaguirre et al. 2011) linked to pre-copulatory barriers (Eizaguirre et al. 2011) and post-copulatory barriers (Eizaguirre et al. 2012a; Kaufmann et al. 2015). Furthermore, lake fish harbor a more diverse parasite community and higher parasite load than river fish resulting in a clear signs of local adaptation of hosts to parasite communities (Eizaguirre et al. 2012b; Kurtz et al. 2004).

Coming from winter conditions experimental males were kept individually in 16 L tanks under winter conditions (8:16 h; 6 °C) and then transferred to spring conditions (12:12 h; 12 °C) for 10 days. Thereafter, in late spring, they were transferred to summer conditions (16:8 h; 18 °C) for the remainder of the experimental period and were provided with nesting material and stimulated to build nests (Sommerfeld et al. 2008). Experimental males and females were kept separately throughout the study and were brought into summer conditions as needed to ensure a continuous availability of fish. Fish were fed with live food (chironomid larvae, glass worms and *Artemia*) twice a day.

### Preference tests

**Figure 1:**
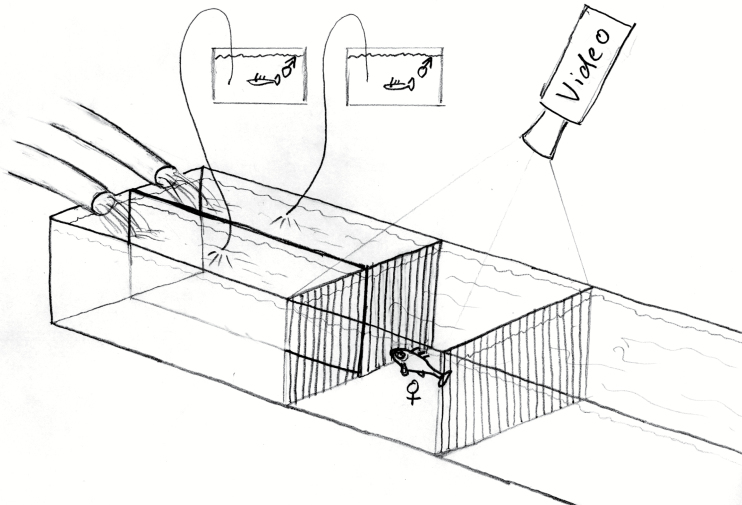
Flow channel design. A gravid female stickleback was placed in the flow chamber that was fed by 2 water columns, to each of which stimulus water was continuously added to the constant water current. Fish were able to freely investigate the composition of water for 2 periods of 300 s each, with spatial reversal of water sources at halftime to control for side effects. Their choice between the front quarters of the chamber was video-recorded from above (Drawing by M.M.).

Female olfactory preference was tested in a flow channel (Milinski et al. 2005; Milinski et al. 2010; Reusch et al. 2001a; Sommerfeld et al. 2008). Female sticklebacks that were ripe for spawning were placed in a flow chamber (Figure 1) that was fed by 2 water columns, to each of which stimulus water (1 L during 600 s) was continuously added, under conditions of laminar flow as described. Fish were able to freely investigate the composition of water in the 2 halves of the chamber for 2 periods of 300 s each, with spatial reversal of water sources at halftime to control for side effects. Their choice in the chamber was video-recorded from above. There were lines drawn on the screen of the monitor, by which it was divided either in halves or further in front and back quarters. If the 2 sources were equally attractive, the fish should spend an equal period of time (i.e. 300 s) with each source of stimulus. Odor preference as determined in the flow channel set-up reliably predicts mate choice (supplementary information of Milinski et al. 2005).

We tested female choice for 2 male conditions: 1) males kept under spring conditions with no nests but developing breeding coloration. Those males are known to release the validation factor but no MHC signal (Milinski et al. 2010); 2) males in summer condition, maintaining (gluing) their nests, i.e., emitting the validation factor as well as their natural MHC signal (Milinski et al. 2010). Male water was collected from the middle of the tank for males with no nest (spring males) and 5 cm above the nest for the “summer males”. Summer males were visually stimulated with a gravid female in a separate tank for 5 min and watched for gluing activity for 15 min after the female stimulus had been removed. Only males that glued their nest within these 20 min were used for the experiments on that day.

Females were transferred in the flow channel using a glass pipe filled with water to reduce disturbance (Milinski and Bakker 1990), and were allowed to acclimate for 5 min. After this time period, we started video recording and waited for the female to cross the middle of the tank within a maximum of 2 min. Once the female had crossed the middle of the tank, an additional 2-min acclimatisation period began. This procedure removed the potential for experimenter bias as to when to add the stimuli. At the end of the second acclimatisation period, a red diode was switched on (only to be seen in the video recording) and stimulus water was added continuously by a pump to each arm of the flow channel in two 5-min experimental periods separated by a 2-min break (neutral water). The stimuli were reversed after the break, controlling for potential female side preference. Female preference was measured from the video recording as the time the tip of her snout spent in each of the 2 front quarters of the flow channel’s test chamber (Milinski et al. 2010). Preference tests were only counted if the female spawned within 24 h after the preference test, in which case the choice in the flow channel reliably predicts mate choice (see electronic supplementary information in Milinski et al. 2005).

### Experiment 1 and control experiment: female preference in presence of only the male validation factor

We tested a potential population specificity of the validation factor by giving female sticklebacks the choice between odor cues of sympatric and allopatric males. For direct comparisons, a female from the lake and a female from the river were sequentially in separate tests given the choice of the olfactory cues of the same pair of males: a lake male from the lake population and a male from the river population, the first being sympatric for the lake female, the second being allopatric for her, vice versa for the river female, for which the lake male was allopatric and the river male sympatric. In order to ensure that the males were only releasing the validation factor, we used synthesized MHC-ligand peptides (see Derivation of MHC peptides section). MHC-ligand peptides are involved in female mate choice and are known to provide information about the individual’s composition of MHC alleles (Milinski et al. 2005). In order to distinguish males that were only releasing the validation factor (see preference tests), we used 4 MHC-ligand peptides. In the second phase of this experiment, the same females were subsequently given the simultaneous choice between their sympatric male’s water supplemented with either: 1) a mixture of 4 MHC-ligand peptides in solvent “wild-type peptides” (Milinski et al. 2005), or 2) solvent only (control experiment). In the presence of the male validation signal, the female would be expected to prefer the side of the flow channel supplemented with MHC peptides, as this mimics a complete male signal (MHC-ligand peptide signal plus validation factor). Without the validation signal, peptide supplementation has no effect (Milinski et al. 2010) and the female would lack a preference. The 2 mate choice tests were spaced a minimum of one hour apart as established (Milinski et al. 2010). If enough ripe females were available, or if a female spawned in between tests, a new female was used per test. Only mate choice tests where males were confirmed as described above to only release the validation factor were used (*n* = 12 per population). Males were only used once.

### Experiment 2: female preference in presence of both the validation factor and the MHC signal

Mate choice was also tested using males, which maintained (glued) their nests: those males are known to release both the MHC signal and the validation factor (Milinski et al. 2010). Eight male pairs (lake and river)—the same as used in Experiment 1—built nests within 3 weeks under summer conditions. Four male pairs of the original 12 took longer than 5 weeks to synchronize the maintenance of a nest and therefore were not included. In order to increase the sample size, one additional lake and one river male pair that had not been used in Experiment 1 was included, resulting in a total of 9 male pairs. Again, 2 preference tests were conducted per male pair, one with a lake and one with a river female. New females were used for each test.

### Derivation of MHC peptides

We used 4 MHC-ligand peptides with the following sequences: SYIPSAEKI, SFVDTRTLL, ASNENMETM, and AAPDNRETF (Milinski et al. 2005; Milinski et al. 2010). Peptides were chemically synthesized, purified, verified by mass spectroscopy (MALDI-TOF), and dissolved in phosphate-buffered saline (PBS) as described (Milinski et al. 2005).

### MHC genotyping

To establish the MHC class IIB genotypes of the fish, we used the reference strand-mediated conformation analysis method (RSCA) following the protocol developed for 3-spined sticklebacks (Lenz et al. 2009). Genomic DNA was extracted from the tip of a single dorsal spine from each individual fish. We amplified the exon 2 of MHC class IIB genes, which encodes the immunologically relevant peptide-binding groove of the MHC molecule. For simplicity, we refer to different sequence variants as alleles, although they may originate from different recently duplicated loci and are thus paralogs (Lenz et al. 2009).

### Statistical analyses

Female preference, i.e. the time the female spent in both 5-min intervals in the quarter of the test tank where a specific male odor arrived, was added up and considered as a single variable (thus controlling for side effects). Because the total time spent in the choice area (the 2 front quarters of the flow channel chamber) varied between tests, we calculated the proportion of time that females spent on each front quarter of the flow channel (Rafferty and Boughman 2006, Eizaguirre et al. 2011). The data did not deviate significantly from a normal distribution (Kolmogorov–Smirnov, Experiment 1: population choice, *n* = 24, KS = 0.49, *P* = 0.967; validation factor, *n* = 28, KS = 0.67, *P* = 0.754; Experiment 2: population choice, *n* = 17, KS = 0.51, *P* = 0.959), justifying the use of parametric tests.

Female preference for sympatric male odor was tested against the expectation of no choice (50% of the time) with a paired *t*-test. Deviation from the no-choice expectation would indicate that the females are able to discriminate between odors. Female choice for sympatric males was also tested using an exact binomial test. Paired *t*-tests and exact binomial tests were performed using combined and split data (split by female population of origin). Female preference for the peptide-supplemented side (Experiment 1) was checked with a paired t-test against the no-choice expectation (50%) using the split dataset. Power analysis was performed following Cohen in G*Power 3 (Faul et al. 2009) to test for “no preference”.

We tested for difference in individual MHC allele diversity between lake and river fish populations using a one-way Anova with individual MHC diversity as dependent variable and habitat type as independent variable. In order to investigate the role of MHC in mate choice, we performed the calculations under the assumptions of a mixed effect model where the predicted individual MHC diversity in the offspring (Reusch et al. 2001a) was used as a dependent variable, and female choice (MHC of the chosen male vs. MHC of the rejected male), habitat of origin of the female, and their interaction were used as independent variables. MHC diversity predicted for the offspring potentially produced with each of the 2 males offered was calculated as the sum of alleles of both possible mates, divided by 2, further corrected by the amount of shared alleles. This number is to be compared with the optimal number of MHC alleles found in the female’s habitat. The predominant genotype in a specific population which has an intermediate number of alleles (e.g. Reusch et al. 2001a) can be shown experimentally to have the highest resistance against local parasites thus carries the optimal number of alleles (e.g., Wegner et al. 2003; Woelfing et al. 2009). For the model, female identity was set as random factor in order to determine with which male her offspring would have an MHC diversity closest to her population’s optimum?

## RESULTS

### Experiment 1 and control experiment: female preference in presence of only the validation factor

To disentangle the effects of the male validation factor from the effects of the MHC signal in assortative mating, we used sticklebacks from the lake and the river and performed multiple flow channel experiments solely based on olfactory cues.

#### Control experiment

**Figure 2:**
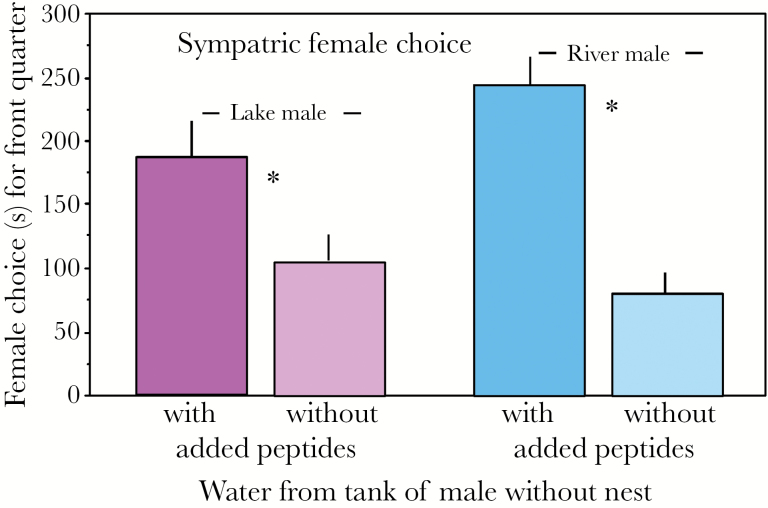
Control experiment—proving that pre-nesting males do not send the MHC signal yet: Lake (pink) and river (blue) females were each presented with the choice between water from a sympatric male (which had not build a nest) and had its water supplemented with MHC peptides and solvent (with peptides, darker color) and only solvent (without peptides, lighter color).Both lake (*n* = 12) and river females (*n* = 12) significantly preferred water from males which was supplemented with synthetic MHC peptides, confirming that the males of either population released only the validation factor and no MHC signal. Although statistics were performed on proportions, for better visualization we the time spent by the females in the front quarters of the flow channel. Means and SE are shown. *P < 0.05.

We established that, under spring conditions, male sticklebacks from either origin only release the validation factor but not the MHC signal. Females were exposed to the water of one male either spiked with MHC-ligand peptides or not. We found that both lake and river females significantly preferred water from males only when it was supplemented with synthetic MHC peptides (Paired *t*-test, lake: df = 14, *P* = 0.034; river: df = 12, *P* = 0.003, Figure 2). In the absence of the validation factor, no preference is expected for peptide-spiked male water (Milinski et al. 2010); likewise, if MHC signals were present in male water, the added peptides would have caused the spiked water source to be rejected because a super-optimal mate would be mimicked (Milinski et al. 2005). Thus, we have shown that both lake males and river males send a validation factor that is appreciated by their sympatric females.

#### Experiment 1

**Figure 3:**
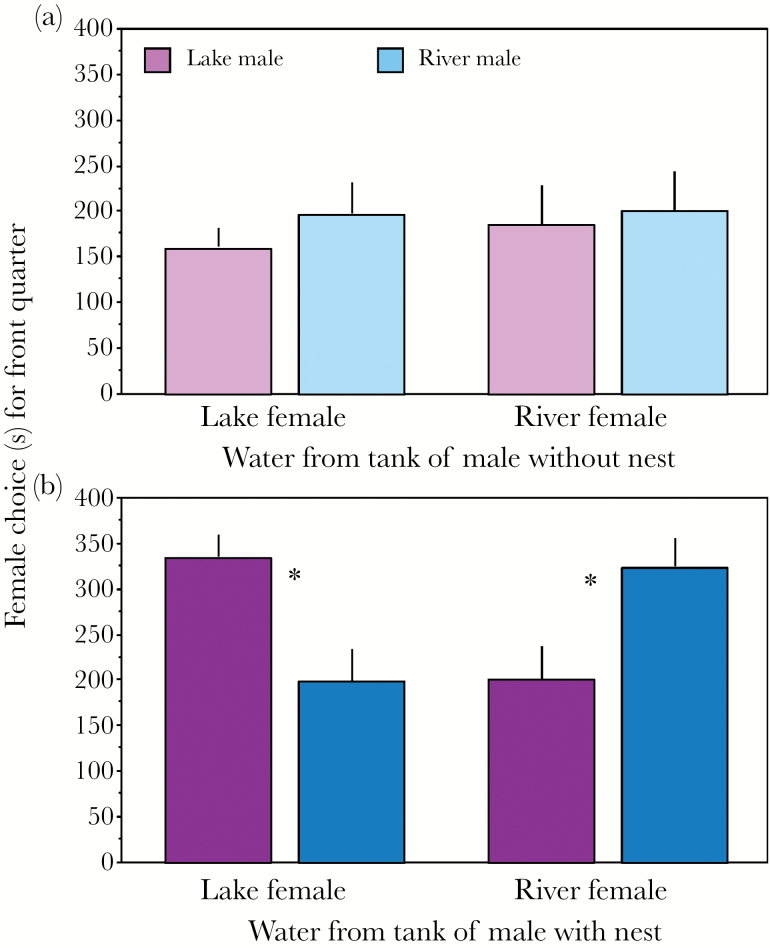
Experiment 1. Females prefer sympatric male odor occurs only in the presence of MHC signal. Each bar represents the time spent in each of the 2 front quarters of the flow channel (in seconds) during olfactory mate choice experiments. Females from either lake or river chose between odor from a lake (pink) and a river male (blue), tested in: (a) The presence of only the validation factor (light colors) (*n* = 24); (b) The presence of both the validation factor and MHC signal (dark colors) (*n* = 17). Although statistics were performed on proportions, for better visualization we depicted the actual time the females spent in the front quarters of the flow channel. Means and SE are shown. *P < 0.05.

Is the validation factor habitat-specific? We had shown previously (Milinski et al. 2010) that water containing only the validation factor is already attractive for gravid females. In the present experiment females that were given the choice between the incomplete (i.e. validation factor only) odor of males from the same and a different habitat were attracted to both odors, but exhibited no preference (no nest, Figure 3a; paired *t*-test, df = 23, *P* = 0.760). This was also the case when the data was split according to female habitat of origin (paired *t*-test, lake: df = 11, *P* = 0.573; river: df = 11, *P* = 0.962). Female choice analyzed with a binomial test also revealed no preference, (Binomial test, combined data, *n* = 11/24, *P* = 0.83; split data, binomial test, lake: *n* = 5/12, *P* = 0.774; river: *n* = 6/12, *P* = 1). Thus, we can conclude that the validation factor is not habitat-specific.

To confirm that the obtained negative results are biologically relevant, we estimated the potential effect size *d* in a power analysis. The power for a paired *t*-test with our data (sample size 24, critical 2-tailed α-level 0.05 and effect size 0.85 [Cohen 1988] was 95.8%; hence, our study had enough power (>80%) to find a significant preference for the maleness validation signal of sympatric males, if it was indeed habitat-specific. Our finding thus corresponds to a “proof” of the null-hypothesis that the validation factor does not offer a habitat-specific clue for mate choice.

### Experiment 2: female preference in presence of both the male validation factor and MHC signal

Using the same male fish as in the previous experiment, we found that females preferred sympatric males when the males were kept under summer conditions and maintained their nest, and thus released both the validation factor and the natural MHC signal according to (Milinski et al. 2010). This was demonstrated by the significant interaction between female origin and male origin on the proportion of time spent in the front quarters of the flow channel (*F*_1,16_ = 19.00, *P* < 0.001): female of lake origin spent proportionally more time on the side of the lake male, while females of river origin favored males of river origin (Figure 3b). The origin of the male was also a significant factor, when data where split for female origin (lake female, *F*_1, 7_ = 9.91, *P* = 0.007; river female, *F*_1,8_ = 9.09, *P* = 0.008).

**Figure 4:**
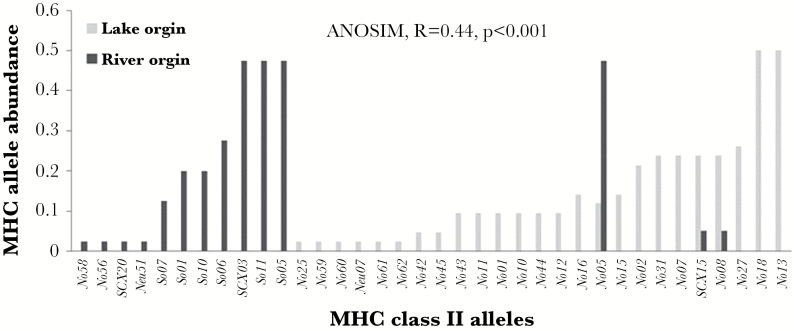
MHC allele pools differ between lake and river populations. As there is locus duplication, abundances are used as proxy for frequencies. The abundances are calculated as the number of times an allele is found in the population divided by the number of fish genotyped (GPS lake, *N* = 42, MA-River, *N* = 40).

Those results suggest that the MHC signal itself contains the population-specific signature and that mate choice favors the maintenance of a locally adapted MHC gene complex. This implies that it is the nature of MHC alleles, which underlies the assortative mate choice decisions of female sticklebacks. Sequence analysis of MHC class II β alleles (Supplementary Material Table S1) not only confirmed that fish of lake origin harbored a higher individual diversity than those from the river (*Z*: −3.558; *P* < 0.0001; lake (mean ± SD): 3.60 ± 0.74, river 2.82 ± 1.11), but also indicated that the pools of MHC alleles are different (ANOSIM, *r* = 0.44, *P* < 0.001, Figure 4).

**Figure 5:**
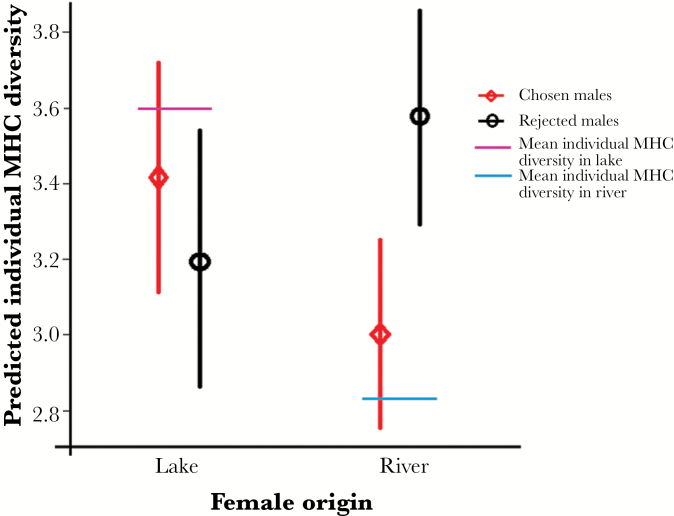
Predicted outcome of mate choice by females from lake and river between water from the tank of a lake and a river males that sent both the validation factor and the MHC odor signal depicted in Figure 3b. Predicted mean ± SE number of individual MHC class II B diversity in potential offspring with respect to MHC diversity of chosen (red) and rejected males (black), as a function of female origin from lake or river (*N* = 11). Individual MHC diversity in the offspring was predicted by calculating the potential combination of the female’s MHC alleles with each of the males’ sets (lake and river) of MHC alleles to be compared with the mean number of MHC alleles found in the female’s habitat of origin, lake or river. Preferred males were those with which, in combination with her own MHC genotype, the female would produce offspring with an individual diversity closer to the mean diversity (horizontal bars, pink for lake, blue for river) of the female’s population of origin (red points for chosen, black points for rejected males). See text for statistics.

Moreover, a significant interaction was revealed between female origin and the origin of the chosen male on the combined number of individual MHC class II β alleles of the offspring as calculated for either chosen or rejected pair combination, (*F*_1,10_ = 5.29; *P* = 0.04): a female significantly preferred the male with which, in combination with her own MHC genotype, she would produce offspring with an individual MHC diversity closer to the mean individual MHC diversity of the female’s population of origin (Figure 5; red points for chosen, black points for rejected males). This demonstrates that a female prefers the male that complements her MHC alleles in such a way that the gamete combination comes closer to the mean individual MHC diversity of her own population, resulting in her choosing the male from her own population. She thus mates assortatively with regards to the MHC. No further factor is needed to allow for assortative mate choice.

## DISCUSSION

Determining the role of sexual selection has become central to our understanding of ecological speciation (Boughman 2002). In particular, a challenge is to identify the ecologically relevant true magic traits, which ensure synergistic action of divergent selection and non-random mating (Servedio et al. 2011).

By using an already optimally MHC-fitting male for a female, we add a mix of 4 synthesized MHC peptide ligands to its natural signal. We used this method in the present study to prove that an experimental male does not send the MHC signal. If there would be a preference for the “same habitat” male, the habitat cannot have been detected from the MHC signal but from something else. There was no habitat related preference. The preference for the 4 peptides’ side, however, proved that the male’s signal contained the validation factor, otherwise the peptides would not have been validated. This provided an experimental proof that the validation factor is not habitat-specific.

Only when presented with the full male olfactory signal, MHC plus validation factor, female sticklebacks chose to mate assortatively with regards to their population of origin. Precisely, a female preferred the male that offered MHC alleles that, combined with her own alleles, would lead to offspring whose MHC is closer to the optimum of the female’s population of origin. This was achieved only by males of her own habitat. Thus, female sticklebacks rely on olfactory cues encoded by the polymorphic MHC genes to select a sympatric male.

Specifically, our findings indicate that the same mechanism that is used to optimize MHC diversity within a population (Reusch et al. 2001a) also leads to assortative mating when females are confronted with the odor from sympatric and allopatric male sticklebacks. Hence, the information contained in peptides released from peptide/MHC complexes, i.e., the MHC-dependent odor signal, is decoded and employed for both purposes. Owing to the differences in MHC allele diversity that exist between the river and lake populations, disassortative mate choice would lead to offspring possessing a non-optimal MHC diversity and the “wrong” types of alleles (Eizaguirre et al. 2009a; Woelfing et al. 2009). For example, if a river female (low MHC allele diversity) chose a lake male, her offspring would have a higher diversity than the mean river population. In contrast, if lake females (high MHC allele diversity) chose river males, their offspring would have a lower combined number of alleles as compared to the lake population mean. Both choices would be sub-optimal and produce less resistant offspring in the female’s habitat. Hence, in order to produce offspring with a mean individual MHC diversity approaching that of the parental population, females choose males that offered the respective complementary MHC alleles. We show here that this is accomplished only when the males originate from the same population as the female (Figure 5). Thus, female sticklebacks employ MHC-dependent olfactory signals to select mates with which they can achieve a habitat-specific MHC gene structure that optimally protects their offspring against local parasites.

Why is the optimum higher in the lake? A host’s overall investment into the adaptive immune system and adaptations to the local parasite fauna and its diversity are important factors. If intra-individual MHC diversity is the result of a trade-off between ensuring efficient presentation of pathogen-derived peptides and some selective force acting against high MHC diversity (e.g. T-cell repertoire depletion, necessity to ensure a high level of antigen presentation or risk of autoimmune diseases), individuals with a MHC diversity just high enough to present peptides of locally abundant parasites and pathogens efficiently will be selected. Low intra-individual MHC diversities may therefore be stable in populations, whose individuals are predominantly challenged by a small pool of pathogens or parasites, which is relatively stable over time. River sticklebacks are challenged by a less diverse parasite fauna than lake sticklebacks. This may explain the finding that the average number of MHC alleles in river sticklebacks is lower than that of lake sticklebacks (Milinski 2006; Woelfing et al. 2009).

Although we cannot exclude the presence of an undetected additional hypothetical olfactory factor that is indeed habitat-specific and could be emitted together with the MHC signal, the choice of population-specific MHC allelic complements is sufficient to allow for habitat-specific assortative mating. Our results suggest that the male validation factor likely signals species-identity to females, whereas the collections of MHC-ligand peptides—as a molecular mirror image of the functional diversity of polymorphic MHC genes represent olfactory signatures of different populations of the same species (Boehm and Zufall 2006).

Even though the ability of female sticklebacks to discriminate against heterospecifics is known (Boughman 2001; Boughman et al. 2005; Kozak and Boughman 2009; Rundle et al. 2000), our study is one of the few that have aimed at dissecting the possible cues involved in such a process. A mechanism of mate choice based on MHC genes as proposed here is also compatible with the use of sequential strategies between species, which aid in female discrimination to strengthen sexual isolation (Kozak et al. 2013). There is considerable debate about the role of sexual selection in driving speciation with numerous theoretical papers published in favor or against (Maan and Seehausen 2011; van Doorn et al. 2009). Empirical evidence supporting the role of sexual selection in driving speciation is accumulating and correlative links between various traits under sexual selection and speciation have been identified (reviewed Maan and Seehausen 2011). As suggested in the present study, scent has been shown to have an important role in premating isolation and thus speciation in a number of species (Smadja and Butlin 2009). There is little evidence on the candidate genes involved in speciation. The present study suggests the role of MHC genes as a true “magic trait” with considerable effect size and proposes a potential mechanism by which MHC drives habitat-specific assortative mate choice, local adaptation and ultimately speciation.

## SUPPLEMENTARY MATERIAL

Supplementary data are available at *Behavioral Ecology* online.

## FUNDING

This work was supported by the Max Planck Society. C.E.’s work is partly supported by DFG grant EI 841/4-1 and EI 841/6-1.

## Supplementary Material

Supplementary_table
