# Supplementary material for: Mate choice in sticklebacks reveals that immunogenes can drive ecological speciation
Source: Behav Ecol. 2017 Jun 1;28(4):953–61. doi: 10.1093/beheco/arx074 (PMC5873247; doi:10.1093/beheco/arx074)
Supplement: Supplementary_table [file arx074_suppl_supplementary_table.doc]

**Table S1:** Amino acid sequences of all MHC class II β (exon 2) present in the genotyped fish. Dots represent amino acids identical to the reference sequence, here designated Neu07.

10 20 30 40 50 60 70

....|....|....|....|....|....|....|....|....|....|....|....|....|....|

**Neu07** **EFIRSSYFNKKEDTRFSSSVGKFVGFTERGVKNAEYWNSNPSYLSAMRAQKEGYCLNSIQIDYTAALTKS**

**Neu51** **.....V.Y..L.F...............Y..R..AA..ND..K....K....V....HVPGY.R......**

**No01**  **.Y.D.YF...L.L...............Y..R......N........K....V....HVPVY.SN.....**

**No02**  **.Y.D.YF...L.L...............Y..G......N........K....V....HVPVY.SN.....**

**No05**  **...D.Y....L.Y...............Y..R......NDA.F.........V....HVPVY.NNV....**

**No07**  **.Y.D.Y.Y..L.L...............Y..R......ND..K....K....V....NV..KHDN.....**

**No08**  **.Y...Y.Y..L.F...............Y.........ND..I....K.........N.....NNV....**

**No10**  **.Y.D.Y.Y..L.Y.........................ND..KV...K........H.............**

**No11**  **.....V.Y..L.F...............Y.........ND..KV...K........HN............**

**No12**  **............................Q...I.AN..KDA.F....K....V....HVPVY........**

**No13**  **.Y..........................Q.........ND..K....K....V...HN...K.DN.....**

**No15**  **.Y.D.Y.Y..L.Y.........Y........RI.AA..NDA.F..GEK....A...HN..NW.NNM....**

**No16**  **.Y...Y.Y..L.Y.........Y.....Y.....AA..N...I..RAK....A...HN.....NNM....**

**No18**  **.....Y.Y..L.F...............Y..R......NDA.L..GEK....V...HN...W.NNM....**

**No25**  **.Y.E.Y.Y..L.F...............Y..........D..K.........V....HVPVY.SN.....**

**No27**  **.Y.D.Y......F...............Q..RI.AA..NDA.F..GEK....A...HN..NW.NNM....**

**No31**  **...Q........................Q...Y.AN..KDA........W..V....HV.S..NNI....**

**No42**  **.Y...Y.Y..L.L.........Y.....Y.....AD..KDA.E....K....V...HN............**

**No43**  **.Y.E.Y.Y..L.F...............Y..R.......D..K.........A....HVPVY.SN.....**

**No44**  **.Y.D.Y......F.........Y.....Q..RI.AA..NDA.F..GEK....A...HN..NW.NNM....**

**No45**  **.Y.D.Y....L.Y...............Y..R......N........K....V....N...K.DN.....**

**No56**  **.....V.Y..L.Y.........................ND..KV...K........HN............**

**No58**  **.....Y.Y..L.F...............Y..R......NDA.L....K....V...HN...W.NNM....**

**No59**  **...D.Y....L.Y...............Y..R......NDA.F.........V....HVPVY.SN.....**

**No60**  **.Y.Q.Y.Y..L.Y...............Y.........NDA.L....K....AV...HVPVY.NNM....**

**No61**  **...D.Y....L.Y.........Y.........I.AD..N...L..GEK....V...HN...W.NNI....**

**No62**  **.Y...Y.Y..L.Y.........Y.....Y..RI.AA..N...I..RAK....A...HN.....NNM....**

**SCX03** **.....Y......................Q..R..AA..N...I..N.K....V....HV....NNI....**

**SCX15** **.Y.D.Y....L.Y...............Y..R......NDA.I..GEK........HN..NW.NNM....**

**SCX16** **...D.Y....L.Y.........Y.....Y.........N...L..GEK....V...HN..NW.NNM....**

**SCX20** **.Y.D.Y.Y..L.L.........Y.....Y.........N...L..GEK....V...HN..NW.NNM....**

**So01**  **.Y.D.Y..........................I.AD..KDA......K....A...HN............**

**So05**  **..........L.Y...............Y..R......NDA.L....K....V....H...E.NNM....**

**So06**  **.Y.....Y..L.Y...............Y.........KDA.F....K....AV..HNV..K.DNM....**

**So07**  **.Y.....Y..L.Y...............Y.........NDA.F....K....AV..HNV..K.DNM....**

**So10**  **.Y.Q.Y.Y..L.L...............Y..R..AA..N......RAK....V....N...K.DN.....**

**So11**  **.Y..........................Q...F.AA..N...I..RAK....V....HVPVY.NNI....**
